# Wire Laser Metal Deposition Additive Manufacturing of Duplex Stainless Steel Components—Development of a Systematic Methodology

**DOI:** 10.3390/ma14237170

**Published:** 2021-11-25

**Authors:** Amir Baghdadchi, Vahid A. Hosseini, Maria Asuncion Valiente Bermejo, Björn Axelsson, Ebrahim Harati, Mats Högström, Leif Karlsson

**Affiliations:** 1 Department of Engineering Science, University West, 461 86 Trollhättan, Sweden; vahid.hosseini@hv.se (V.A.H.); asun.valiente@hv.se (M.A.V.B.); ebrahim.harati@itwwelding.com (E.H.); mats.hogstrom@hv.se (M.H.); leif.karlsson@hv.se (L.K.); 2 Alfa Laval Tumba AB, 147 80 Tumba, Sweden; bjorn.axelsson@alfalaval.com; 3 ITW Welding AB, 433 25 Partille, Sweden

**Keywords:** additive manufacturing, duplex stainless steel, laser metal deposition, methodology, mechanical properties, microstructure characterization

## Abstract

A systematic four-stage methodology was developed and applied to the Laser Metal Deposition with Wire (LMDw) of a duplex stainless steel (DSS) cylinder > 20 kg. In the four stages, single-bead passes, a single-bead wall, a block, and finally a cylinder were produced. This stepwise approach allowed the development of LMDw process parameters and control systems while the volume of deposited material and the geometrical complexity of components increased. The as-deposited microstructure was inhomogeneous and repetitive, consisting of highly ferritic regions with nitrides and regions with high fractions of austenite. However, there were no cracks or lack of fusion defects; there were only some small pores, and strength and toughness were comparable to those of the corresponding steel grade. A heat treatment for 1 h at 1100 °C was performed to homogenize the microstructure, remove nitrides, and balance the ferrite and austenite fractions compensating for nitrogen loss occurring during LMDw. The heat treatment increased toughness and ductility and decreased strength, but these still matched steel properties. It was concluded that implementing a systematic methodology with a stepwise increase in the deposited volume and geometrical complexity is a cost-effective way of developing additive manufacturing procedures for the production of significantly sized metallic components.

## 1. Introduction

Additive manufacturing (AM), commonly known as 3-dimensional (3D) printing, refers to manufacturing processes that fabricate parts by adding layers on top of each other [1,2]. It has opened doors for the fabrication of near-net-shape components with low waste of materials, customized features, and complex geometries. Laser Metal Deposition with Wire (LMDw) is an AM technique in which the wire is melted with a laser beam and deposited layer-by-layer (bead-by-bead) to build a component. In LMDw, the implementation of a laser beam in combination with an advanced controlling system allows appropriate monitoring and control of the process [3,4]. In addition to the low cost of raw material (the wire) [5] and high material usage efficiency (up to 100%), LMDw has provided the opportunity to reach comparatively high productivity [6] and is, therefore, suitable for the production of full-size near-net-shape parts for industrial applications. Preheating the wire feedstock using the hot-wire technique can be used to further increase the deposition rate of LMDw [3], thereby improving productivity.

Duplex stainless steels (DSS) with a ferritic-austenitic microstructure offer high mechanical properties and excellent corrosion resistance [7]. They have, therefore, received much attention in different industries, including the petrochemical, oil and gas, pulp and paper, desalination, and pollution control industries [8]. The optimum properties of DSS are achieved for approximately equal fractions of ferrite and austenite. In laser AM of DSS, the often high cooling rate can cause an excessive amount of ferrite and nitride formation. In addition, the nature of AM processes, which consist in the deposition of layers on top of each other, leads to the formation of secondary austenite in reheated layers, which can degrade both mechanical and corrosion properties [9,10]. Nitrogen loss, moreover, restricts sufficient austenite formation and can consequently affect phase balance in DSS [11,12]. Therefore, controlling the chemical composition and thermal cycles is vital in AM of DSS to ensure a desirable microstructure. Post-process treatment, particularly post-heat treatment, can also be used to achieve a balanced ferritic-austenitic microstructure and improve the properties of additively manufactured DSS.

In recent years, there have been several studies on AM of DSS. Using powder bed fusion, researchers have fabricated DSS parts with selective laser melting (SLM) [13,14,15,16]. In all cases, an excessive amount of ferrite was a problem in the microstructures of the additively manufactured parts, and a post-heat treatment was necessary to balance ferrite and austenite fractions. Wire-arc additive manufacturing (WAAM) of DSS has also attracted widespread interest due to the affordable equipment and its high deposition rate [17,18,19,20,21,22,23,24,25,26]. Zhang et al. [22] observed an unbalanced microstructure with a high fraction of austenite in WAAM of super DSS. In another study, it was reported the WAAM of 2209 DSS wire led to the formation of more than 70% austenite [23]. In addition to the formation of a high austenite fraction, WAAM lacks feature resolution and bead morphology control; consequently, the manufactured parts need significant machining [27].

However, the potential of using LMDw in AM of DSS still remains largely unknown. In addition, although there have been many studies on AM of DSS, no systematic and generally applicable approach to the fabrication of real components has yet been addressed. Recently, Valiente et al. [4] studied the production of a single-bead wall DSS by LMDw as an initial stage of the work presented in more detail here. They produced a single-bead wall and studied the microstructures in both as-deposited and heat-treated conditions. In this study, a systematic four-stage methodology was developed and applied to the production of a cylinder intended for industrial applications. The methodology included the deposition of a single-bead pass, a single-bead wall, a block, and finally a cylinder, and the aim was to systematically, step by step, increase the geometrical complexity and size of the manufactured parts. For each step, the microstructure was evaluated, and the chemical composition was analyzed. In addition, for the last two stages, the mechanical properties in as-deposited and heat-treated conditions were studied. This approach made it possible to systematically evaluate and control the effects of an increment both in the amount of deposited material and in the heat treatment on the microstructures and properties. Finally, a cylinder with an inner diameter of 160 mm and a thickness of 30 mm was successfully produced and extensively tested.

## 2. The Systematic Four-Stage Methodology

By extending AM processes beyond rapid prototyping and into the manufacturing of final products, manufacturing constraints should be less severe and design freedom could be expanded [28]. A four-stage methodology, therefore, was developed to produce a cylinder, intended for industrial applications, by LMDw.

**Figure 1:**
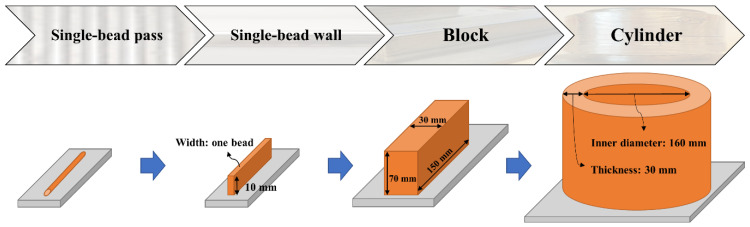
The four-stage methodology applied to the production of a DSS near-net-shape component by LMDw.

The outline of this methodology, which demonstrates how both the volume of deposited material and geometrical complexity increase through the stages, is presented in Figure 1. The aim and approach of each stage, as well as the testing strategy including the evaluation performed in each stage, are described in Table 1.

The results of the first and second stages were investigated in [3,4], and full details of the studies of the block produced in stage 3 and the cylinder produced in stage 4 will be within the scope of separate publications. In this paper, the application of the systematic methodology is presented, and examples of microstructures, chemical compositions, and mechanical properties leading up to the cylinder as the final components are presented.

## 3. Experimental

### 3.1. Materials

In all four stages, the substrates for material deposition were 10-mm thick duplex stainless steel plates of type 2205 (UNS S322059). A solid wire duplex stainless steel of type 2209 (EN ISO 14343-A: G 22 9 3 N L) with a 1.2 mm diameter was used as the feedstock in all stages. A wire batch with a slightly different chemical composition was used in the fourth stage. The chemical compositions of the substrate and wires are presented in Table 2.

### 3.2. Laser Metal Deposition with Wire Setup

**Figure 2:**
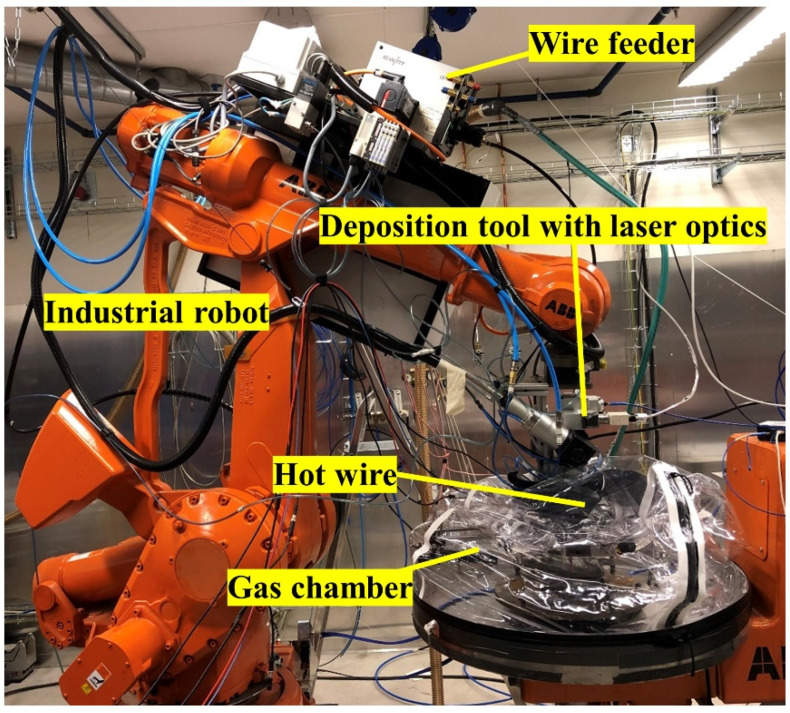
Laser Metal Deposition setup.

A picture of the LMDw setup [4] is shown in Figure 2. It includes a 6 kW Ytterbium-doped fiber laser, a 6-axis robot from ABB, Sweden, a deposition tool with laser optics, a wire feeder, a control system, and actuators. A programmable logic control (PLC) was used to control the deposition during the fabrication of components. The wire feed system was also equipped with hot-wire technology, in which an electrical current is used to resistively pre-heat the wire and thereby increase the deposition rate. An electrical power source regulated the current and the voltage for pre-heating and was controlled by online monitoring. The hot-wire control system aims to maintain a specific resistance to ensure a stable metal transfer, good wettability, and dimensional control. More details about the LMDw setup can be found in [3].

The additively manufactured components were built with this setup for all four stages. In the following section, the procedure used in the LMDw of the cylinder is explained in more detail. 

The additively manufactured components were built with this setup for all four stages. A description of the procedure used in the LMDw of the cylinder is explained in more detail in Section 4.1. 

### 3.3. Heat Treatment

The additively manufactured block and cylinder were investigated in as-deposited and heat-treated conditions. The heat treatment was conducted in a furnace equipped with a thermocouple to measure and control the heat treatment temperature. Heat treatment was performed for 1 h after reaching 1100 °C in an air atmosphere, and the sample was then cooled by water quenching. The heat treatment procedure was selected to achieve a balanced content of ferrite and austenite [29], to ensure the dissolution of nitrides, and to avoid sigma formation.

### 3.4. Testing and Characterization

Microstructures of the single-bead pass, single-bead wall, block, and cylinder were studied by light optical microscopy. Cross-sections of additively manufactured parts were prepared for optical microscopy as presented in [30] and etched with modified Beraha. A Zeiss Axio Imager.M2m optical microscope was used to study the microstructures of the single-bead pass, single-bead wall, block, and cylinder. Phase fraction measurements were performed by image analysis (IA) via the open-access ImageJ software. 

The chemical compositions of the additively manufactured components in the last two stages were analyzed by optical emission spectroscopy (OES). Nitrogen content, in addition, was measured by combustion analysis in all stages.

For evaluation of mechanical properties in the last two stages, tensile and Charpy impact toughness tests were performed on samples machined from the block and the cylinder in as-deposited and heat-treated conditions. The tensile tests were performed at room temperature according to EN ISO 6892-1. For the block, four tensile samples were extracted only parallel to the deposition direction while for the cylinder, three samples were machined, both parallel and perpendicular to the deposition directions. Charpy impact testing was performed at −10 °C according to EN ISO 148-1. For both block and cylinder, impact test specimens were extracted parallel and perpendicular to the deposition directions. The results are presented as the average of two and four tested samples for the block and cylinder, respectively.

## 4. Results

In this chapter, the outputs of the four-stage methodology are introduced, and it is shown how the knowledge gained from each stage is implemented in the following stage. Then, microstructures and results from chemical analysis, as well as mechanical testing, are presented.

### 4.1. Material Produced by the Four-Stage Methodology

**Figure 3:**
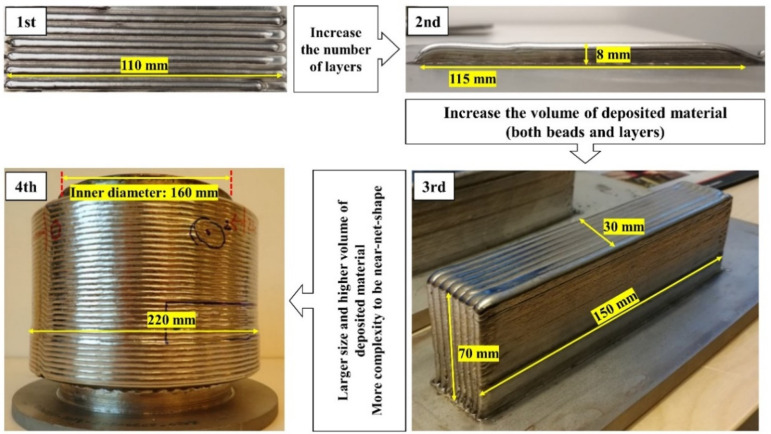
Four-stage methodology in LMDw of DSS cylinder. First: single-bead pass (1 bead, 3.5 g); second: single-bead wall (10 beads, 35 g); third: block (2.5 kg); and fourth: cylinder (>20 kg). The deposition volume and complexity were increased stepwise to reach the final component.

The laser metal deposited duplex stainless steel parts, produced by LMDw in the four stages, are presented in Figure 3.

**Figure 4:**
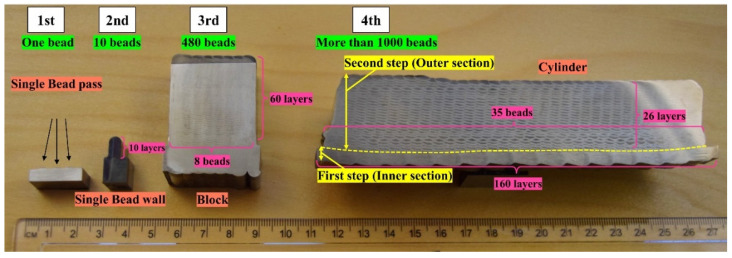
Cross-sections of single-bead pass, single-bead wall, block, and cylinder. Increase in the number of deposited beads from the first stage to the fourth one.

Visual inspection and light optical microscopy of cross-sections of the additively manufactured single-bead pass, single-bead wall, block, or cylinder (Figure 4) showed no signs of lack of fusion and only a few very small pores. 

**Stage 1**: In the first stage, several single-bead passes were deposited with a length of 110 mm and a weight of around 3.5 g. Online data monitoring during the deposition revealed that the implementation of a pre-heated wire allows fine-tuning of the heat input. Wire pre-heating, in addition, improves process stability and minimizes the risk of formation of lack of fusion defects. More details about the single-bead deposits can be found in [3]. 

**Stage 2**: In the second stage, a single-bead wall was produced. Here, the height of the AM part increased to around 8 mm by the deposition of 10 layers on top of each other, and the deposit weight was 35 g. Successful control of the melt pool volume by adjustment of wire feed speed, wire preheating, welding speed, and laser power made it possible to produce a wall with vertical flat surfaces free from visible defects between layers. Chemical analysis, however, showed that some nitrogen loss occurred [4]. 

**Stage 3**: In the third stage, a block was produced with a significantly larger number of beads and layers. In this stage, the block consisted of 60 layers with 8 parallel beads in each layer—480 beads altogether. The weight of the additively manufactured block increased more than 70 times compared to the 2nd stage and was near 2.5 kg. As for the single-bead wall, some nitrogen loss occurred, but the nitrogen content was sufficient to reach a suitable phase balance after heat treatment. Mechanical testing, moreover, revealed that the LMDw block fulfilled the required properties. On the other hand, heat accumulation elevated the temperature of the manufactured part during LMDw. As a consequence, several stops had to be made during the building of the block, which increased the production time and disrupted the continuity of the manufacturing. Therefore, it was found that a method of cooling the component during production was essential to avoid excessive temperature build-up and provide stable production conditions.

**Figure 5:**
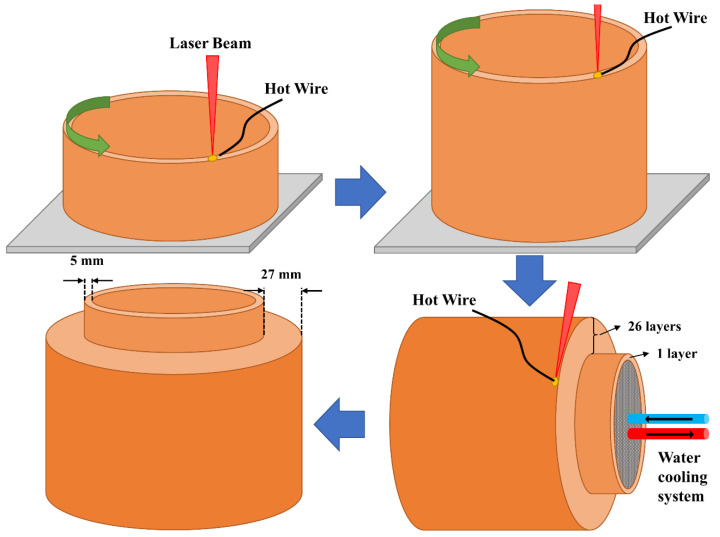
Steps in additive manufacturing of the cylinder. The circular deposition was used to build the inner section, which was used as the substrate for the LMDw of the second (outer) section. The first section also provided the possibility of using a water-cooling system. The deposition of the second section was perpendicular to the first deposition direction.

**Stage 4**: Finally, in the fourth stage, as illustrated in Figure 5, the laser metal deposition of the cylinder was conducted with two sections: an inner section and an outer one. First, an inner tube-shaped section, acting as the substrate for the outer section, was deposited on a 2205 DSS plate substrate. For the inner tube section, after each complete circular layer, the substrate shifted in the Z direction for the deposition of a new layer for all 160 layers. In addition to acting as the substrate for the deposition of the second section, the inner tube section allowed the implementation of an internal water-cooling system during the LMDw of the outer section. The water-cooling system worked by circulation of the cold water inside the inner section of the cylinder. In this way, the part could be produced continuously, eliminating the need for unwanted cooling stops. Before depositing the outer section, the inner section was turned 90 degrees. Fabrication was performed by the deposition of 35 passes parallel to each other in 26 layers. The process parameters used for the production of the LMDw cylinder are listed in Table 3.

The cylinder was fabricated by deposition of more than 1000 beads and the weight of the deposited material was more than 20 kg. The significantly higher volume of deposited material, greater complexity, and a continuous process were three main goals in the production of the cylinder. This was successfully achieved by employing the systematic four-stage methodology. 

### 4.2. Microstructure

In this section, a comparison of the microstructures of the single-bead pass, single-bead wall, block, and cylinder in different locations and conditions are presented.

#### 4.2.1. Last Bead and Reheated Bead Microstructures

In this section, the DSS microstructures of the last bead and the microstructures after reheating, due to deposition of the following beads and layers, are presented. 

**Figure 6:**
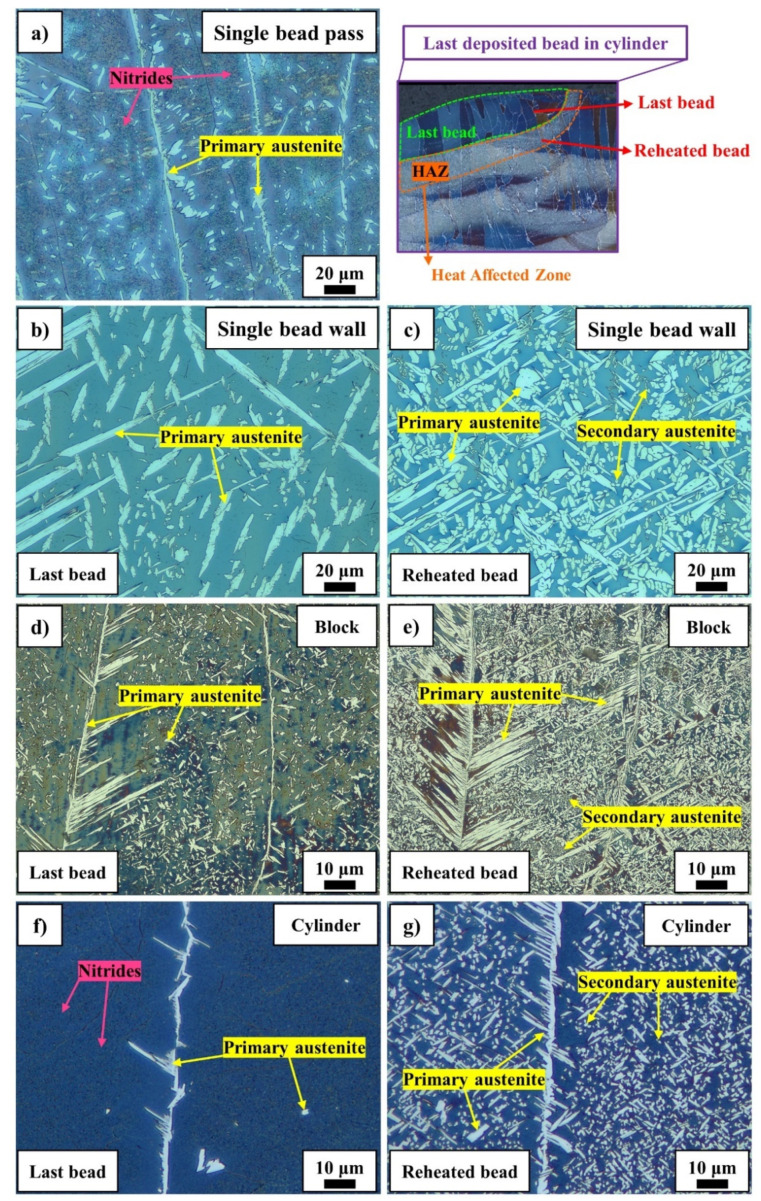
Light optical microscopy of LMDw components. The microstructures of a single-bead pass and of last deposited and underlying beads in a single-bead wall, block, and cylinder. Samples were etched with modified Beraha, showing ferrite as the dark and austenite as the bright phase. Growth of primary austenite and formation of secondary austenite are seen in the reheated beads.

As illustrated in Figure 6, the last bead microstructures of 2205 duplex stainless steel consist of grain boundary, Widmanstätten, and intragranular austenite on the ferritic matrix. In the last deposited bead, all austenite grains are primary since they were formed during solidification and have not experienced any additional reheating cycles. According to the formation mechanism, two types of austenite can form in DSS—primary austenite and secondary austenite. Duplex stainless steels solidify fully ferritic and primary austenite forms on cooling in a solid-state transformation of ferrite to austenite at ferrite/ferrite grain boundaries, as well as inside the ferrite grains [31,32]. Secondary austenite forms during additional reheating cycles. In ferritic regions, clusters of small black precipitates can be observed which are interpreted as chromium nitrides [11,33,34]. Although the last deposited bead of the single-bead wall, block, and cylinder had highly ferritic microstructures, reheating increased austenite fractions considerably. In the last deposited bead of single-bead walls, the austenite fraction was 23 ± 3%, which increased to 40 ± 4% in the reheated bead due to deposition of the next bead. The increment of austenite fraction was due to the growth of primary austenite and the formation of secondary austenite. In the production of the block, the last bead and the reheated bead had 16 ± 2% and 52 ± 3% austenite, respectively. The last deposited bead in the cylinder had a nearly fully ferritic microstructure with only 2% austenite found at the ferrite/ferrite grain boundaries. Reheating increased the austenite fraction up to 33 ± 3% as the result of the growth of grain boundary austenite, as well as the formation of Widmanstätten and intragranular secondary austenite.

#### 4.2.2. As-Deposited and Heat-Treated Bulk Microstructures

**Figure 7:**
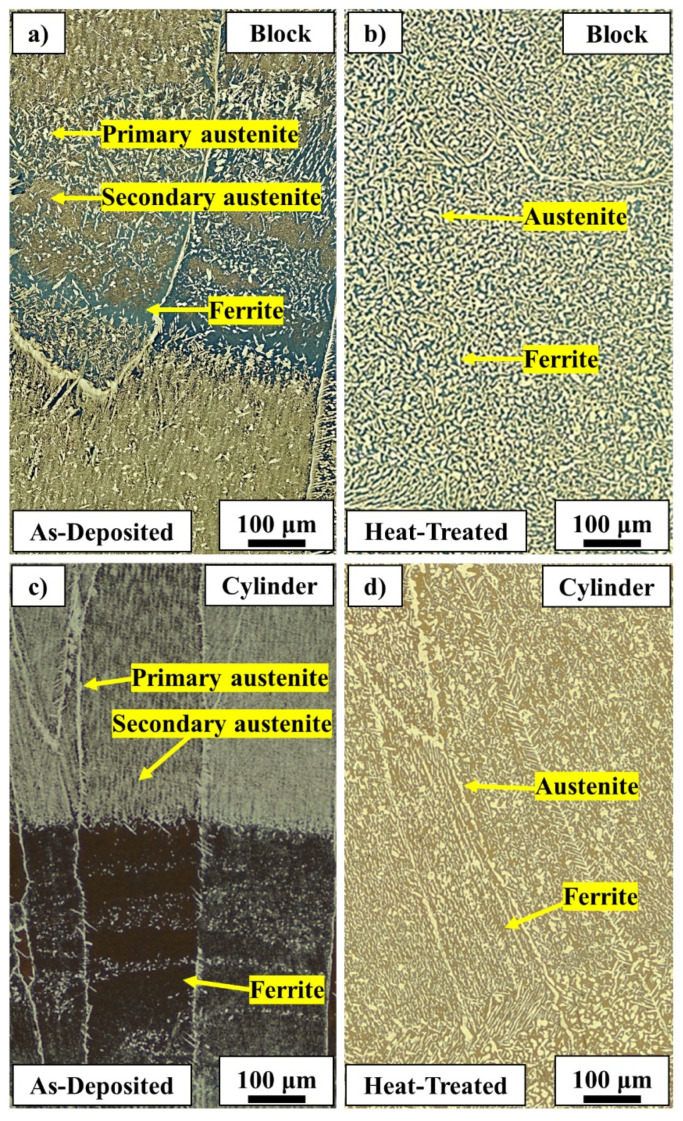
Light optical micrographs of LMDw block and cylinder microstructures. (**a**,**c**): As-deposited microstructures including highly ferritic areas and regions with a high fraction of austenite. (**b**,**d**): Heat-treated microstructure with a homogeneous distribution of ferrite (dark phase) and austenite (bright phase). Heat treatment homogenized the microstructure and balanced ferrite and austenite fractions.

The microstructures of the laser metal deposited block and cylinder in as-deposited and heat-treated conditions are shown in Figure 7. In the as-deposited condition, the microstructures were inhomogeneous (see also Figure 6) and included various regions such as highly ferritic areas, regions with a high fraction of secondary austenite, and areas with a combination of both primary and secondary austenite. The inhomogeneity of the as-deposited microstructure was most pronounced in the cylinder. After heat treatment at 1100 °C for 1 h, however, the microstructure was homogeneous with relatively equal fractions of ferrite and austenite. The austenite fractions were 51± 2% and 50± 1% for the heat-treated block and cylinder, respectively. Heat treatment caused the growth and coarsening of both primary and secondary austenite.

**Figure 8:**
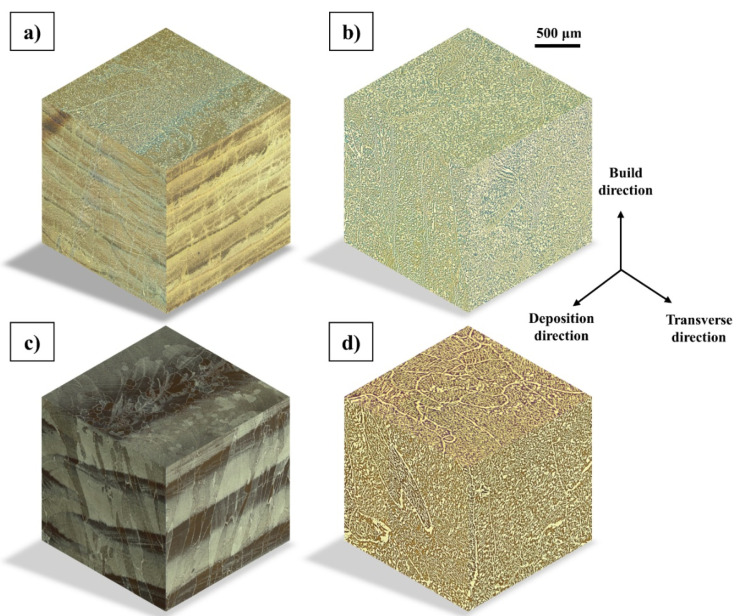
The 3D microstructures of laser metal deposited block and cylinder produced by light optical microscopy. (**a**) As-deposited block, (**b**) heat-treated block, (**c**) as-deposited cylinder, and (**d**) heat-treated cylinder. An inhomogeneous microstructure in as-deposited condition and homogeneous distribution of ferrite (dark) and austenite (bright) after heat treatment is shown.

The 3D microstructures of the LMDw block and cylinder in both as-deposited and heat-treated conditions are shown in Figure 8. For both the block and the cylinder, the repetition of highly ferritic and highly austenitic layers shows the inhomogeneous microstructures in the as-deposited condition. After heat treatment, however, there is no layer-by-layer microstructure, and a homogeneous distribution of ferrite and austenite is seen in both block and cylinder.

### 4.3. Chemical Analysis

The nitrogen content measurements of the four stages, as presented in Table 4, revealed that nitrogen loss occurred during duplex stainless steel LMDw regardless of the size and shape of the fabricated parts. It can also be seen that, by increasing the volume of deposited material, the nitrogen content reached the stable amount of 0.11 wt.%. Optical emission spectroscopy analyses of the block and cylinder in Table 5, however, show that the contents of other elements were similar after stages three and four with differences directly related to the composition of the two wire batches used (Table 2). 

### 4.4. Mechanical Properties

**Figure 9:**
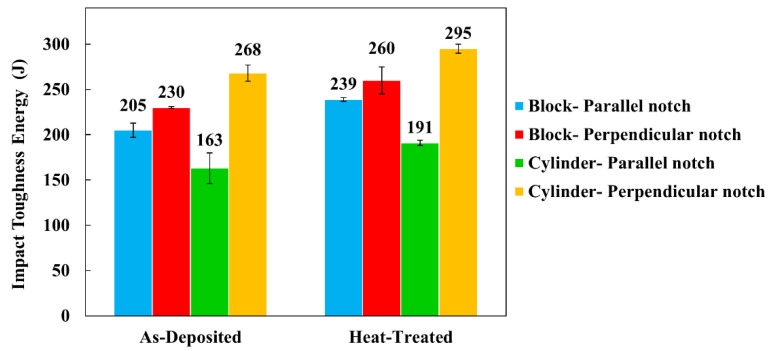
The Charpy impact toughness energy of DSS block and cylinder. Samples with notch perpendicular and parallel to the deposition direction. A notch perpendicular to deposition direction and heat treatment resulted in higher impact energies.

The results of Charpy testing at −10 ℃ for samples with a notch both parallel to and perpendicular to the deposition direction in both as-deposited and heat-treated conditions are presented in Figure 9. For both block and cylinder, the heat-treated specimens had higher impact toughness energies compared to the as-deposited condition. In addition, in both block and cylinder, the samples with a notch perpendicular to the deposition direction had higher impact toughness energies in as-deposited and heat-treated conditions. 

**Figure 10:**
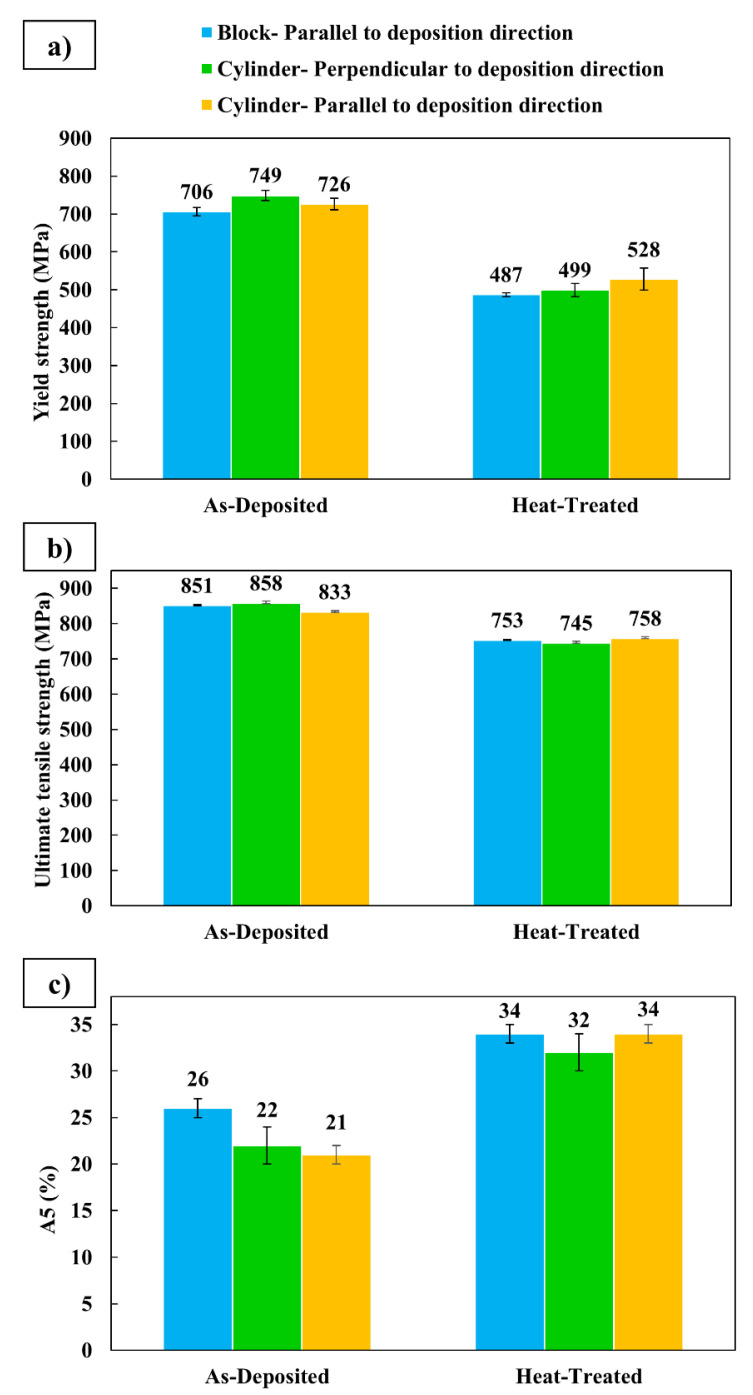
Tensile test results. (**a**) Yield strength, (**b**) ultimate tensile strength, and (**c**) elongation of block and cylinder in as-deposited and heat-treated conditions.

The results of tensile testing, including average yield strength, ultimate tensile strength, and elongation for the block and cylinder in as-deposited and heat-treated conditions, are presented in Figure 10. In tensile tests, samples parallel to the deposition direction of the block and specimens parallel and perpendicular to the deposition direction of the cylinder had almost similar tensile behavior. For both block and cylinder, the average yield strengths were between 706 and 749 MPa in as-deposited condition. After heat treatment, however, the average fell to 487–528 MPa. The average ultimate tensile strengths were between 833 and 858 MPa and between 745 and 758 MPa in as-deposited and heat-treated conditions, respectively. The average elongation increased from 21–26% to 32–34% after heat treatment.

### 4.5. Thermodynamic Calculations

**Figure 11:**
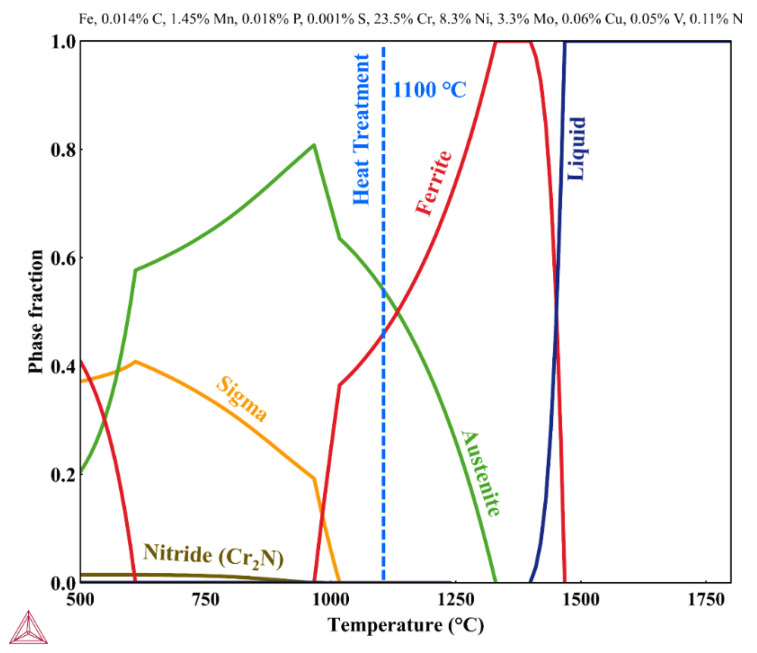
Phase diagram calculated with Thermo-Calc for laser metal deposited DSS components with a nitrogen content of 0.11 wt.%.

Nitrogen loss affects the phase balance of duplex stainless steels and lowers the austenite formation start temperature in these alloys. Therefore, the equilibrium phase diagram for the chemical composition of the laser metal deposited components was calculated by Thermo-Calc to find the appropriate heat treatment temperature. As can be seen in Figure 11, at around 1100 °C, the equilibrium ferrite and austenite fractions are approximately 50%.

## 5. Discussion

The stepwise four-stage methodology made it possible to achieve a stable and consistent LMDw process to successfully manufacture a high-quality near-net-shape cylinder with properties suitable for industrial applications.

In this section, the quality, nitrogen loss, microstructures, and mechanical properties of the material produced by LMDw in as-deposited and heat-treated conditions are discussed. After that, the applicability of the four-stage methodology is commented on.

### 5.1. Deposit Quality

One of the main challenges in the laser deposition, and the AM in general, of metallic materials is minimizing the occurrence of defects as these tend to act as crack initiation sites [35]. For example, experience from laser cladding has shown that avoiding formation of defects such as pores, cracks, and poor/lack of fusion can be a challenge [36]. However, previous studies using laser metal deposition with stainless steel wire have demonstrated that high-quality products can be fabricated without defects such as porosity, cracks, and a lack of fusion [5,37]. This is in line with the findings of the present study, where the only defects found were a few small pores.

### 5.2. Nitrogen Loss

Chemical analysis in all stages revealed that nitrogen loss happened during the LMDw of duplex stainless steel components. In the production of the cylinder, the nitrogen content was 0.11 wt.% as for the block and single-bead wall [4]. Hosseini et al. [17] suggested that it is expected to have more nitrogen loss in AM compared to welding. However, the nitrogen loss in the laser metal deposited cylinder is lower than the nitrogen loss reported in laser welding of DSS [12]. 

Since nitrogen is a strong austenite former, its loss can significantly affect the phase balance and properties of DSS components. In addition to nitrogen, nickel content also influences the phase balance and, subsequently, the properties of DSS products [38,39]. Therefore, based on thermodynamic calculations for the chemical composition of the LMDw components, the heat treatment temperature of 1100 ℃ was selected in which fractions of ferrite and austenite were around 50%. As results showed, despite the nitrogen loss, the combination of the increased nickel content of the wire and the heat treatment successfully produced a well-balanced microstructure with nearly equal fractions of ferrite and austenite.

### 5.3. Microstructure

In the manufacturing of duplex stainless steel components, microstructure control is of great importance since the best combination of mechanical properties and corrosion resistance usually comes by approximately equal fractions of ferrite and austenite [40,41].

#### 5.3.1. As-Deposited

Rapid cooling limits austenite formation during the manufacturing of DSS components as illustrated by the microstructures of last deposited beads for single-bead pass, single-bead wall, block, and cylinder in Figure 6. This behavior was also observed in laser welding of duplex stainless steel [42]. Due to the rapid cooling, ferrite also became supersaturated by nitrogen, and consequently, nitrides formed in highly ferritic regions on cooling and reheating. In the single-bead wall, the heat conduction was only possible in the build direction, while in the block, the heat conduction occurred in two directions which resulting in a higher cooling rate. In the cylinder, the significantly higher cooling rate due to the implementation of a water-cooling system led to the negligible formation of austenite in as-deposited condition. However, additional reheating and cooling cycles provided sufficient time at elevated temperatures for nitrogen diffusion and austenite formation via the growth of primary grain boundary, Widmanstätten, and intragranular austenite and the formation and growth of secondary austenite [43,44,45].

The addition of layers on top of each other and multiple reheating cycles resulted in a complicated but periodically repetitive microstructure which has also been observed in multipass welding [45,46]. The repetitive microstructure of duplex stainless steel during additive manufacturing was also observed by Posch et al. [19] and Lervag et al. [20] in WAAM of DSS. 

#### 5.3.2. Heat-Treated

Heat treatment homogenized the microstructure and balanced the ferrite and austenite fractions with approximately equal fractions of these phases. It should be noted that, as shown in Figure 11, the heat treatment temperature was selected to give slightly higher austenite than ferrite content to compensate for the fact that the material is unlikely to fully reach the equilibrium condition during heat treatment. Hengsbach et al. [16] also found that heat treatment can significantly promote austenite formation in additively manufactured DSS components. 

Moreover, heat treatment effectively as expected dissolved nitrides since ferrite and austenite are the only stable phases at 1100 °C (Figure 11). Heat treatment also caused a coarsening of the grain boundary, the Widmanstätten, and especially the intragranular austenite. In addition to the growth after heat treatment, intragranular austenite had a globular morphology, contrary to the angular morphology found in as-deposited condition. This is in line with the results of heat-treated duplex and super duplex stainless steel [29,47]. 

### 5.4. Mechanical Properties

In tensile testing, the similar behaviors of specimens oriented parallel to and perpendicular to the deposition direction demonstrate the isotropy of the tensile properties in this additively manufactured component. This was in contrast with the results of Zhang et al. [23] who reported up to 11% anisotropy of tensile properties in WAAM with a 2209 DSS wire. 

The decrease in strength and increase in elongation after heat treatment is in the line with observations for selective laser-melted 2205 DSS powder [14]. In their study, 45% and 24% reductions in yield and ultimate tensile strengths, after 1 h heat treatment at 1050 ℃, were observed. It should be noted that the tensile properties are high even after heat treatment. This is attributed to the fine microstructure of the additively manufactured components, which is retained after heat treatment.

Heat treatment increased impact toughness energy up to 17% since, as explained in the previous section, it homogenized the microstructure and removed nitrides. Specimens from both block and cylinder with the notch perpendicular to the deposition direction had up to 39% higher impact toughness energies than those with a parallel orientation (Figure 9). This is attributed to the orientation of grain boundary austenite along the build direction as the result of the epitaxial growth of ferrite. The epitaxial growth of ferrite grains along the build direction was also observed by Hengsbach et al. [16] in AM of duplex stainless steel by SLM. The anisotropy of mechanical properties parallel and perpendicular to the deposition direction was also reported by Zhang et al. [22] in WAAM of super DSS. 

### 5.5. Additive Manufacturing of Duplex Stainless Steel Components 

**Figure 12:**
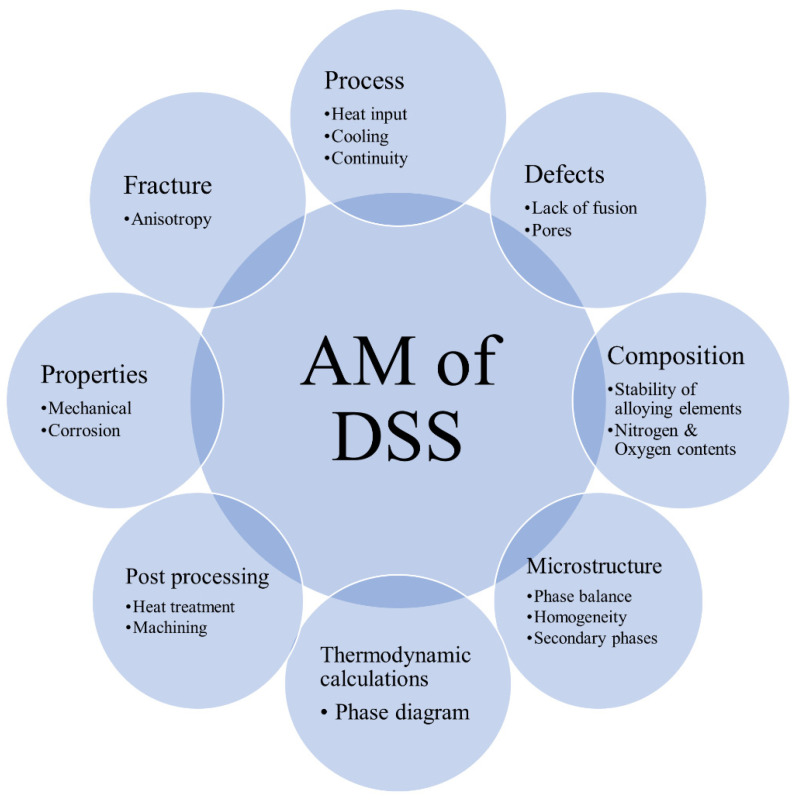
Important factors in additive manufacturing of duplex stainless steel components. The properties of DSS components are dictated by their microstructure, which is controlled by the chemical composition and thermal cycles. Reaching required properties and avoiding defects require implementation and control of appropriate process parameters and the use of suitable feedstock material.

It has been demonstrated in this and other studies that additive manufacturing can be used to build DSS components, although achieving a desired microstructure and properties can be a challenge, as discussed in the introduction [13,14,15,16,17,18,23,24,25,26]. Figure 12 summarizes some important factors during production, the tools available to predict the resulting microstructure, and important properties that need to be controlled.

Several factors are important in AM of DSS, as listed in Figure 12. To begin with, from the viewpoint of the process, it is important to design a system that provides appropriate cooling to avoid heat accumulation and allow a continuous process and high productivity. Heat input, size and geometry of the component, and material grade all are important in controlling the temperature cycle including maximum allowable temperature and appropriate cooling rates [48]. Another challenge in AM is minimizing the occurrence of defects such as porosity, cracking, and lack of fusion, as defects will deteriorate the mechanical properties [35]. Another factor is the component chemical composition, particularly oxygen and nitrogen contents, since oxygen affects the ductility and toughness, and nitrogen influences phase balance and therefore properties [49,50]. The most important factor is the microstructure, governing properties, which was investigated in the present study as presented in this and earlier papers [3,4]. Thermodynamic calculations are useful to predict the formation of phases, phase balance, and the effects of heat treatment. From both mechanical and corrosion resistance perspectives, approximately equal fractions of ferrite and austenite usually present the optimum functionality of DSS [41]. The homogenizing of the microstructure and prevention of the formation of detrimental secondary phases are actions that should be taken to reach high-quality products [51]. After the production of the parts, post-process treatments are often necessary to further improve the properties. The most important is a post-heat treatment, which was implemented to homogenize the microstructure, remove secondary phases, and balance the ferrite and austenite ratio. Finally, the mechanical properties and corrosion resistance of DSS components determine the applicability of AM in the fabrication of these alloys. Testing is therefore needed to evaluate and verify the mechanical properties and the corrosion resistance and to judge component functionality. 

### 5.6. Four-Stage Methodology

A four-stage systematic methodology was developed for the manufacturing of DSS components by LMDw (Table 1) and applied to the production of a near-net-shape cylinder intended for industrial applications. In this study, from the first step to the final component, not only did the number of beads and the volume of deposited material increase significantly but the geometry of the manufactured parts also became more complex. Employing the four-stage methodology, however, made it possible to successfully fabricate a high-quality cylinder.

Concerning process and control aspects from the first stage to the fourth, when increasing complexity of the manufactured part, the importance of controlling heat input and wire pre-heating increased, since these determined the shape and volume of the melt pool and, therefore, the resulting geometry of deposited layers. In addition, by increasing the size of the fabricated part, the implementation of a cooling system became essential to prevent an increase in temperature that would require “cooling stops”, which, in turn, decrease productivity.

Similar complicated and periodically repetitive inhomogeneous as-deposited microstructures were observed in the last three stages. Mechanical properties of the as-deposited parts produced in the last two stages were on a satisfactory level. However, the inhomogeneity of the as-deposited microstructure made it clear that a post-heat treatment after LMDw of duplex stainless steel components might be needed or might, at least, be beneficial, depending on the product and intended application. A heat treatment homogenized the microstructure and was also, particularly when used in combination with thermodynamical calculations, effective in handling the nitrogen loss during LMDw, aiming at a well-balanced nitride-free ferritic-austenitic microstructure. 

A conclusion from the present study was that a stepwise increase in deposit volume and geometrical complexity is highly recommended when designing an AM production procedure intended for components of significant size. It is a time-efficient way of finding process parameters and identifying potential problems, while wasting only a limited amount of material. The four-stage methodology introduced, although here applied only to one process and one material, is in principle general and can, with modifications to process, component, and material, be applied generally to AM of metallic components. However, further investigations are needed to evaluate the applicability of this methodology with different materials and various processes.

The LMDw components, particularly the cylinder, have mechanical properties well-suited for many industrial applications. However, future testing of properties should include corrosion resistance and fatigue properties, in both as-deposited and heat-treated conditions, to fully define suitable application areas.

## 6. Conclusions

A systematic four-stage methodology for AM of significantly sized metallic components was developed and applied to Laser Metal Deposition with Wire of a near-net-shape duplex stainless steel cylinder intended for industrial applications. In the four stages, single-bead passes, a single-bead wall, a block, and a cylinder were produced, while process parameters and control systems were developed, evaluated, and fine-tuned. The chemical composition, microstructure, and mechanical properties of LMDw components were studied in as-deposited and heat-treated conditions.

1. The implementation of the four-stage methodology made it possible to achieve a stable and consistent LMDw process while, step by step, the volume of deposited material and the complexity of the additively manufactured components increased.
2. Addition of water cooling was found necessary to avoid heat accumulation when increasing component size.
3. The final components were of high quality with no cracks or lack of fusion defects, and only a few small pores were detected.
4. Some nitrogen loss was observed resulting in a content of 0.11% N in the cylinder compared to 0.14% N in the wire.
5. The as-deposited duplex microstructure was inhomogeneous and repetitive and included regions with low and high fractions of austenite. Nitrides were observed in highly ferritic regions.
6. Heat treatment at 1100 °C for 1 h locally and globally homogenized the microstructure, removed nitrides, and balanced the ferrite and austenite fractions. The austenite fractions reached around 50% after heat treatment.
7. Strength and toughness were at a high level, comparable to those of the corresponding steel grade, both as-deposited and after heat treatment. The highest strength was achieved in as-deposited condition with an average yield strength of 749 MPa and average UTS of 858 MPa, and the best toughness and ductility was in the heat-treated condition by an average of 34%. The heat treatment increased the toughness and ductility, while it decreased the strength.

## Figures and Tables

**Table 1:** Aim, approach, and testing strategy of each stage in the four-stage methodology. Y (YES) = test was performed, N (NO) = test was not performed.

**# Stage**	**Component produced by LMDw**	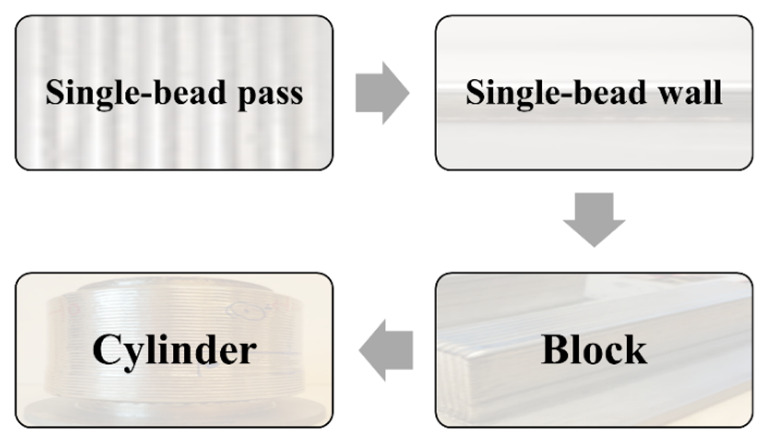	Evaluations							
			Process stability	Bead shape and geometry	Inspection for lack of fusion, porosity, and inclusions	Post-production homogenization heat treatment	Chemical analysis		Microstructure characterization & study phase balance	Mechanical testing: strength and toughness
							Nitrogen measurement	Full chemical analysis		
**1**	**Single-bead pass**	**Aim**: Find a process window, giving a stable process in single-bead deposition. **Approach**: Systematic testing of combinations of parameters such as power, travel speed, wire feed rate, and wire pre-heating.	**Y**	**Y**	**N**	**N**	**Y**	**N**	**Y**	**N**
**2**	**Single-bead wall**	**Aim**: Find a process window, giving a stable process for single-bead wall deposition, control of the geometry and avoiding imperfections. **Approach**: Systematic testing of combinations of control system setting parameters and process parameters such as power, travel speed, wire feed rate, and wire pre-heating.	**Y**	**Y**	**Y**	**N**	**Y**	**N**	**Y**	**N**
**3**	**Block**	**Aim**: Apply learnings from previous stages to the production of a block to find a process window and control settings, giving a stable process and control of the geometry and avoiding imperfections. **Approach**: Systematic testing of combinations of the control system setting parameters and process parameters such as power, travel speed, wire feed rate, and wire pre-heating based on learnings from previous stages.	**Y**	**Y**	**Y**	**Y**	**Y**	**Y**	**Y**	**Y**
**4**	**Cylinder**	**Aim**: Verify developed deposition procedure by the production of a full-size near-net-shape component in the shape of a cylinder with 160 mm inner diameter, 30 mm thickness, and height of 140 mm. **Approach**: Systematic testing of combinations of control system setting parameters and process parameters such as power, travel speed, wire feed rate, and wire pre-heating, based on learnings from the stage.	**Y**	**Y**	**Y**	**Y**	**Y**	**Y**	**Y**	**Y**

**Table 2:** Chemical compositions of the plate and wire (wt.%).

	C	Si	Mn	P	S	Cr	Ni	Mo	Cu	N
Plate	0.016	0.32	1.77	0.027	<0.001	22.77	5.50	3.07	0.21	0.18
Wire-batch 1	0.016	0.45	1.45	0.016	0.001	23.23	8.62	3.29	0.04	0.16
Wire-batch 2	0.013	0.52	1.48	0.018	0.001	23.50	8.35	3.40	0.08	0.14

**Table 3:** LMDw process parameters.

Laser power (W)	3500
Wire feed rate (m/min)	2
Deposition speed (mm/s)	10
Focal length (mm)	300
Wavelength (nm)	1040
Spot size (mm)	3
Shielding gas	Argon
Hot-wire voltage (V)–Average value	1.5 V in the first layer and 1 V in the subsequent layers
Hot-wire current (A)–Average values	~100 A in the first layer and ~70 A in the subsequent layers

**Table 4:** Nitrogen measurement of as-deposited LMDw samples by combustion analysis.

Sample	Single-Bead Pass	Single-Bead Wall	Block	Cylinder
Nitrogen (wt.%)	0.14	0.11	0.11	0.11

**Table 5:** Chemical composition of the additively manufactured components analyzed by optical emission spectroscopy (OES).

	C	Si	Mn	P	S	Cr	Ni	Mo	Cu	V
Block	0.022	0.45	1.46	0.016	0.002	23.22	8.48	3.17	0.04	0.08
Cylinder	0.014	0.48	1.42	0.018	<0.002	23.54	8.30	3.32	0.06	0.05

## Data Availability

The data presented in this study are available on request from the corresponding author.

## References

[1] Haghdadi N., Laleh M., Moyle M., Primig S. (2021). Additive manufacturing of steels: A review of achievements and challenges. J. Mater. Sci..

[2] ASTM (2012). Standard Terminology for Additive Manufacturing Technologies.

[3] Kisielewicz A., Thalavai Pandian K., Sthen D., Hagqvist P., Valiente Bermejo M.A., Sikström F., Ancona A. (2021). Hot-Wire Laser-Directed Energy Deposition: Process Characteristics and Benefits of Resistive Pre-Heating of the Feedstock Wire. Metals.

[4] Valiente Bermejo M.A., Thalavai Pandian K., Axelsson B., Harati E., Kisielewicz A., Karlsson L. (2021). Microstructure of laser metal deposited duplex stainless steel: Influence of shielding gas and heat treatment. Weld. World.

[5] Shaikh M.O., Chen C.C., Chiang H.C., Chen J.R., Chou Y.C., Kuo T.Y., Ameyama K., Chuang C.H. (2020). Additive manufacturing using fine wire-based laser metal deposition. Rapid Prototyp. J..

[6] Kulkarni J.D., Goka S.B., Parchuri P.K., Yamamoto H., Ito K., Simhambhatla S. (2021). Microstructure evolution along build direction for thin-wall components fabricated with wire-direct energy deposition. Rapid Prototyp. J..

[7] Verma J., Taiwade R.V. (2017). Effect of welding processes and conditions on the microstructure, mechanical properties and corrosion resistance of duplex stainless steel weldments—A review. J. Manuf. Process..

[8] Sarlak H., Atapour M., Esmailzadeh M. (2015). Corrosion behavior of friction stir welded lean duplex stainless steel. Mater. Des..

[9] Karlsson L., Arcini H. (2012). Low energy input welding of duplex stainless steels. Weld. World.

[10] Karlsson L. (2012). Welding duplex stainless steels-A review of current recommendations. Weld. World.

[11] Hosseini V.A., Wessman S., Hurtig K., Karlsson L. (2016). Nitrogen loss and effects on microstructure in multipass TIG welding of a super duplex stainless steel. Mater. Des..

[12] Baghdadchi A., Hosseini V.A., Hurtig K., Karlsson L. (2020). Promoting austenite formation in laser welding of duplex stainless steel—impact of shielding gas and laser reheating. Weld. World.

[13] Davidson K., Singamneni S. (2016). Selective Laser Melting of Duplex Stainless Steel Powders: An Investigation. Mater. Manuf. Process..

[14] Papula S., Song M., Pateras A., Chen X.B., Brandt M., Easton M., Yagodzinskyy Y., Virkkunen I., Hänninen H. (2019). Selective laser melting of duplex stainless Steel 2205: Effect of post-processing heat treatment on microstructure, mechanical properties, and corrosion resistance. Materials.

[15] Saeidi K., Kevetkova L., Lofaj F., Shen Z. (2016). Novel ferritic stainless steel formed by laser melting from duplex stainless steel powder with advanced mechanical properties and high ductility. Mater. Sci. Eng. A.

[16] Hengsbach F., Koppa P., Duschik K., Holzweissig M.J., Burns M., Nellesen J., Tillmann W., Tröster T., Hoyer K.P., Schaper M. (2017). Duplex stainless steel fabricated by selective laser melting-Microstructural and mechanical properties. Mater. Des..

[17] Hosseini V.A., Högström M., Hurtig K., Valiente Bermejo M.A., Stridh L.E., Karlsson L. (2019). Wire-arc additive manufacturing of a duplex stainless steel: Thermal cycle analysis and microstructure characterization. Weld. World.

[18] Zhang X., Wang K., Zhou Q., Kong J., Peng Y., Ding J., Diao C., Yang D., Huang Y., Zhang T. (2020). Element partitioning and electron backscatter diffraction analysis from feeding wire to as-deposited microstructure of wire and arc additive manufacturing with super duplex stainless steel. Mater. Sci. Eng. A.

[19] Posch G., Chladil K., Chladil H. (2017). Material properties of CMT—metal additive manufactured duplex stainless steel blade-like geometries. Weld. World.

[20] Lervåg M., Sørensen C., Robertstad A., Brønstad B.M., Nyhus B., Eriksson M., Aune R., Ren X., Akselsen O.M., Bunaziv I. (2020). Additive manufacturing with superduplex stainless steel wire by cmt process. Metals.

[21] Stuzer J., Totzauer T., Wittig B., Zinke M., Juttner S. (2019). GMAW Cold Wire Technology for Adjusting the Manufactured Duplex Stainless Steel Components. Metals.

[22] Zhang X., Wang K., Zhou Q., Ding J., Ganguly S., Grasso M., Yang D., Xu X., Dirisu P., Williams S.W. (2019). Microstructure and mechanical properties of TOP-TIG-wire and arc additive manufactured super duplex stainless steel (ER2594). Mater. Sci. Eng. A.

[23] Zhang Y., Cheng F., Wu S. (2021). The microstructure and mechanical properties of duplex stainless steel components fabricated via flux-cored wire arc-additive manufacturing. J. Manuf. Process..

[24] Wanwan J., Chaoqun Z., Shuoya J., Yingtao T., Daniel W., Wen L. (2020). Wire Arc Additive Manufacturing of Stainless Steels: A Review. Appl. Sci..

[25] Hejripour F., Binesh F., Hebel M., Aidun D.K. (2019). Thermal modeling and characterization of wire arc additive manufactured duplex stainless steel. J. Mater. Process. Technol..

[26] Eriksson M., Lervåg M., Sørensen C., Robertstad A., Brønstad B.M., Nyhus B., Aune R., Ren X., Akselsen O.M. (2018). Additive manufacture of superduplex stainless steel using WAAM. MATEC Web Conf..

[27] Greer C., Nycz A., Noakes M., Richardson B., Post B., Kurfess T., Love L. (2019). Introduction to the design rules for Metal Big Area Additive Manufacturing. Addit. Manuf..

[28] Yang S., Zhao Y.F. (2015). Additive manufacturing-enabled design theory and methodology: A critical review. Int. J. Adv. Manuf. Technol..

[29] Kotecki D.J. (1989). Heat treatment of duplex stainless steel weld metals. Weld. J..

[30] Baghdadchi A., Hosseini V.A., Karlsson L. (2021). Identification and Quantification of Martensite in Ferritic-Austenitic Stainless Steels and Welds. J. Mater. Res. Technol..

[31] Lippold J.C., Kotecki D.J., Sant S. (2006). Welding Metallurgy and Weldability of Stainless Steels. MRS Bull. Res. Soc..

[32] Zhang Z., Jing H., Xu L., Han Y., Zhao L. (2016). Investigation on microstructure evolution and properties of duplex stainless steel joint multi-pass welded by using different methods. Mater. Des..

[33] Kim S.T., Lee I.S., Kim J.S., Jang S.H., Park Y.S., Kim K.T., Kim Y.S. (2012). Investigation of the localized corrosion associated with phase transformation of tube-to-tube sheet welds of hyper duplex stainless steel in acidified chloride environments. Corros. Sci..

[34] Tan H., Wang Z., Jiang Y., Han D., Hong J., Chen L., Jiang L., Li J. (2011). Annealing temperature effect on the pitting corrosion resistance of plasma arc welded joints of duplex stainless steel UNS S32304 in 1.0M NaCl. Corros. Sci..

[35] Shamsaei N., Yadollahi A., Bian L., Thompson S.M. (2015). An overview of Direct Laser Deposition for additive manufacturing; Part II: Mechanical behavior, process parameter optimization and control. Addit. Manuf..

[36] Abioye T.E., Medrano-Tellez A., Farayibi P.K., Oke P.K. (2017). Laser metal deposition of multi-track walls of 308LSi stainless steel. Mater. Manuf. Process..

[37] Xu X., Mi G., Luo Y., Jiang P., Shao X., Wang C. (2017). Morphologies, microstructures, and mechanical properties of samples produced using laser metal deposition with 316 L stainless steel wire. Opt. Lasers Eng..

[38] Kim S.T., Jang S.H., Lee I.S., Park Y.S. (2011). Effects of solution heat-treatment and nitrogen in shielding gas on the resistance to pitting corrosion of hyper duplex stainless steel welds. Corros. Sci..

[39] Muthupandi V., Bala Srinivasan P., Shankar V., Seshadri S.K., Sundaresan S. (2005). Effect of nickel and nitrogen addition on the microstructure and mechanical properties of power beam processed duplex stainless steel (UNS 31803) weld metals. Mater. Lett..

[40] Gunn R.N. (1997). Duplex Stainless Steels: Microstructure, Properties and Applications.

[41] Messer B., Oprea V., Wright A. (2007). Duplex stainless steel welding: Best practices. Stainl. Steel World.

[42] Chaudhari A.N., Dixit K., Bhatia G.S., Singh B., Singhal P., Saxena K.K. (2019). Welding behaviour of duplex stainless Steel AISI 2205: AReview. Mater. Today Proc..

[43] Valiente Bermejo M.A., Hurtig K., Eyzop D., Karlsson L. (2019). A new approach to the study of multi-pass welds-microstructure and properties of welded 20-mm-thick superduplex stainless steel. Appl. Sci..

[44] Ramirez A.J., Brandi S.D., Lippold J.C. (2004). Secondary austenite and chromium nitride precipitation in simulated heat affected zones of duplex stainless steels. Sci. Technol. Weld. Join..

[45] Hosseini V.A., Hurtig K., Karlsson L. (2020). Bead by bead study of a multipass shielded metal arc-welded super-duplex stainless steel. Weld. World.

[46] Valiente Bermejo M.A., Eyzop D., Hurtig K., Karlsson L. (2021). Welding of large thickness super duplex stainless steel: Microstructure and properties. Metals.

[47] Park S., Shin B., Park J., Kim D., Chung W. (2019). Effect of austenite morphology on the electrochemical properties of super duplex stainless UNS S 32750. Int. J. Electrochem. Sci.

[48] DebRoy T., Wei H.L., Zuback J.S., Mukherjee T., Elmer J.W., Milewski J.O., Beese A.M., Wilson-Heid A., De A., Zhang W. (2018). Additive manufacturing of metallic components–Process, structure and properties. Prog. Mater. Sci..

[49] Moon Y.H., Kim H.T., Hur S.D. (1987). Effect of oxygen content on impact toughness of austenitic and duplex stainless steel weld metal. J. Korean Weld. Soc..

[50] Jeon S.-H., Hur D.H., Kim H.-J., Park Y.-S. (2014). Influence of oxygen content on the inclusion formation and pitting corrosion resistance of hyper duplex stainless steels. Mater. Trans..

[51] Karlsson L. (1999). Intermetallic phase precipitation in duplex stainless steels and weld metals: Metallurgy, influence on properties, welding and testing aspects. Weld. Res. Counc. Bull..

